# Cell-Laden Marine Gelatin Methacryloyl Hydrogels Enriched with Ascorbic Acid for Corneal Stroma Regeneration

**DOI:** 10.3390/bioengineering10010062

**Published:** 2023-01-04

**Authors:** Ana L. Alves, Ana C. Carvalho, Inês Machado, Gabriela S. Diogo, Emanuel M. Fernandes, Vânia I. B. Castro, Ricardo A. Pires, José A. Vázquez, Ricardo I. Pérez-Martín, Miguel Alaminos, Rui L. Reis, Tiago H. Silva

**Affiliations:** 1 3B’s Research Group, i3B’s—Research Institute on Biomaterials, Bisodegradables and Biomimetics, University of Minho, Headquarters of the European Institute of Excellence on Tissue Engineering and Regenerative Medicine, AvePark, Parque de Ciência e Tecnologia, Zona Industrial da Gandra, 4805-017 Guimarães, Portugal; 2 ICVS/3B’s—PT Government Associate Laboratory, 4806-909 Braga/Guimarães, Portugal; 3 Group of Recycling and Valorization of Waste Materials (REVAL), Instituto de Investigaciones Marinas (IIM-CSIC), C/Eduardo Cabello 6, CP36208 Vigo, Spain; 4 Group of Food Biochemistry, Instituto de Investigaciones Marinas (IIM-CSIC), C/Eduardo Cabello 6, CP36208 Vigo, Spain; 5 Department of Histology and Tissue Engineering Group, Faculty of Medicine, University of Granada and Instituto de Investigación Biosanitaria (ibs.GRANADA), E18016 Granada, Spain

**Keywords:** marine biomaterials, gelatin, codfish, GelMA, keratocytes, cornea

## Abstract

Corneal pathologies from infectious or noninfectious origin have a significant impact on the daily lives of millions of people worldwide. Despite the risk of organ rejection or infection, corneal transplantation is currently the only effective treatment. Finding safe and innovative strategies is the main goal of tissue-engineering-based approaches. In this study, the potential of gelatin methacryloyl (GelMA) hydrogels produced from marine-derived gelatin and loaded with ascorbic acid (as an enhancer of the biological activity of cells) was evaluated for corneal stromal applications. Marine GelMA was synthesized with a methacrylation degree of 75%, enabling effective photocrosslinking, and hydrogels with or without ascorbic acid were produced, encompassing human keratocytes. All the produced formulations exhibited excellent optical and swelling properties with easy handling as well as structural stability and adequate degradation rates that may allow proper extracellular matrix remodeling by corneal stromal cells. Formulations loaded with 0.5 mg/mL of ascorbic acid enhanced the biological performance of keratocytes and induced collagen production. These results suggest that, in addition to marine-derived gelatin being suitable for the synthesis of GelMA, the hydrogels produced are promising biomaterials for corneal regeneration applications.

## 1. Introduction

The cornea is the outer component of the eye and the first to be in contact with the external environment, being commonly known as the front door of this organ. This avascular and transparent structure has two major functions: protection of the eye from external aggressions [1] and refraction of light (approximately 65% to 80%) together with the lens [2,3]. The human cornea is a multilayered tissue composed of five major layers: an anterior layer called the epithelium, followed by Bowman’s membrane, the stroma, Descemet’s membrane, and a posterior layer known as the endothelium [4,5]. The stroma is the thickest layer, accounting for 90% of corneal thickness. The stromal extracellular matrix (ECM) is composed of a complex array of collagen fibers (mainly types I and V) oriented perpendicularly between layers, sulfated glycosaminoglycans (such as lumican, decorin, biglycan, and keratocan) that control collagen production and assembly, and keratocytes, which are responsible for ECM remodeling [6]. Disruption of stromal homeostasis by trauma, injury, or even infection, can result in irreversible visual impairment, which can culminate in blindness, affecting millions worldwide [7]. Numerous strategies have been used to treat stromal damage as the most common replacement of corneal tissue by a graft from a human donor [8]. In severe cases, the current viable therapeutic approach is the use of corneal prosthetics, such as the Boston KPro [9]. However, both strategies have several drawbacks [10,11], and new therapeutic approaches that can fulfill the stromal features and necessities are urgently needed.

Tissue Engineering (TE) has been used to explore these gaps, and several biomaterials have been studied for corneal stromal applications [12,13]. Several collagen-based biomaterials have been developed during the last years [14,15,16,17,18,19]. However, they are usually based on collagen from mammals or recombinant origin, which brings some drawbacks/concerns related to immunogenic reactions [20,21,22], zoonosis transmission (e.g., bovine spongiform encephalopathy (BSE), transmissible spongiform encephalopathy (TSE), and foot and mouth disease (FMD)) [23] due to infected material. In addition, ethnical constraints (Hindu, Muslim, and Jewish cultures) [24] and even high costs associated with low yields (in the case of recombinant collagen) [25] are also important hurdles. Considering this, marine-origin collagenous materials have gained special attention as a safer alternative since there is no identified zoonotic transmission through the material [26,27,28]. Moreover, the large amount of fish waste produced by fishing industries, which represents up to 70–85% of the total weight of a catch, results in waste or low-value by-products [29]. Thus, fish materials such as skins, scales, and swim bladders have been seen as attractive sources for the extraction of bioactive compounds such as collagen and gelatin, being proposed as an economically and environmentally sustainable alternative. Previous works of our group demonstrated the low immunogenic response [30], biocompatibility, and good potential of marine-origin collagenous materials for TE applications [31,32,33,34,35,36,37].

Despite all the evidence of their application in the TE field, few reports using marine-derived collagenous materials are available for corneal regeneration or replacement. Studies conducted by Lin et al. [38] on acellular tilapia fish scales and by Feng et al. [39] on the lamellar structure of grass carp scales demonstrated good mechanical and optical properties, as well as the promotion of cellular proliferation and migration on these natural biomaterials, thus showing them to be promising scaffolds for cornea regeneration. Additionally, following studies demonstrated that type I collagen matrix derived from the scales of tilapia offers light scatter and transmission close to the human cornea, as well as good biocompatibility in mouse, rat, rabbit, and pig models [40,41,42,43]. Currently, this fish-scale-derived collagen matrix, BioCornea, is undergoing phase I clinical trials [44].

In the present work, gelatin extracted from codfish (*Gadus morhua*) salted skins was used, for the first time, as a building block to produce a bioengineered gelatin methacryloyl (GelMA) hydrogel for corneal stroma regeneration. GelMA has emerged as an attractive material for cell culture because it can be easily synthesized at a low cost while providing cell attachment sites (RGD motifs) that promote cell adhesion, migration, and proliferation. At the same time, it is transparent in the same way as native corneal tissue, and its concentration and degree of functionalization (DoF) can be adjusted to achieve a proper network distribution and stiffness for better biological performance. Herein, we aimed to evaluate the ability of marine-based GelMA hydrogels (mGelMA) to meet the physical and biological features of the corneal stroma, as well as to serve as a drug carrier capable of releasing different molecules of interest. In this context, we selected ascorbic acid (AA) based on its antioxidant character that could help keratocytes resist oxidative stress [45], as well as to stimulate its natural collagen production, as shown by other authors [46,47,48].

## 2. Materials and Methods

### 2.1. Chemical Reagents and Material

Methacrylic anhydride, 2-Hydroxy-4′-(2-hydroxyethoxy)-2-methylpropiophenone (Irgacure 2959), sodium bicarbonate, phosphate-buffered saline (PBS), Dulbecco’s PBS (DPBS), dialysis tubing membrane (12-14 k-CA cut off), bovine serum albumin (BSA), Triton X-100, 4′,6-Diamidino-2-phenylindole dihydrochloride (DAPI) were purchased from Sigma-Aldrich (St. Louis, MO, USA). SYLGARD™ 184 Silicone Elastomer Kit was purchased from Dow Corning (Midland, MI, USA). L-Ascorbic Acid Phosphate Magnesium Salt n-Hydrate was acquired from FUJIFILM Wako Pure Chemical Corporation (Osaka, Japan). Paraformaldehyde (PFA) and TrypLE™ Express (Gibco) were acquired from Thermo Fisher Scientific (Waltham, MA, USA). Collagenase A from *Clostridium histolyticum* (0.223 U/mg) was purchased from Roche Diagnostics (Switzerland). Human corneal keratocytes (HK, P10872), fibroblast medium kit (P60108), and poly-L-lysine (0413) were purchased from Innoprot (Derio, Spain). Primary antibodies, rabbit anti-collagen I (NB600-408), goat anti-collagen V (NBP1-26551), and rabbit anti-lumican (NBP2-76847) were purchased from Novus Biologicals (Littleton, CO, USA) and rabbit anti-decorin (PA5-27370) from Thermo Fisher Scientific (Waltham, MA, USA). Secondary Antibodies, mouse anti-goat IgG-CFL 488 was acquired from Santa Cruz Biotechnology (Santa Cruz, CA, USA) and Alexa Fluor™ 488 donkey anti-rabbit was purchased from Thermo Fisher Scientific. Gelatin from Atlantic codfish skin industrial wastes (*Gadus morhua*) was extracted and characterized as previously described [37].

### 2.2. Synthesis of Marine Gelatin Methacryloyl (mGelMA)

**Figure 1:**
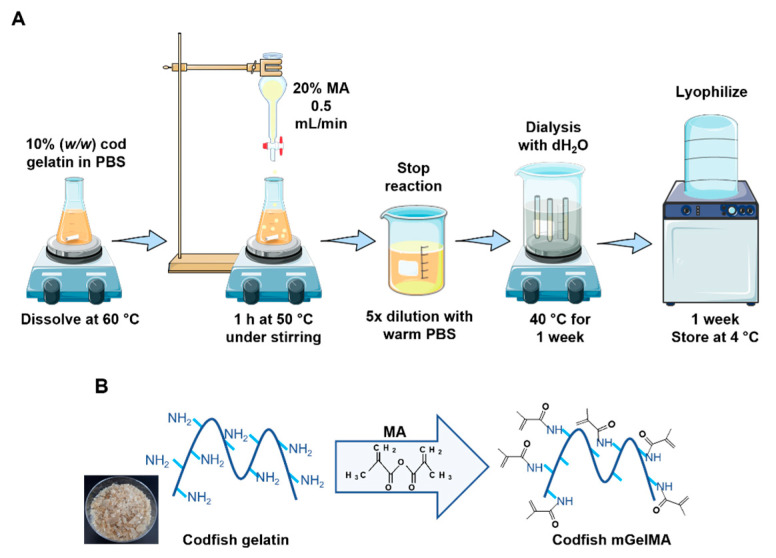
Overview of the mGelMA production process. (**A**) Schematic illustration of the mGelMA production. After gelatin dissolution, the MA (methacrylic anhydride) was added very slowly under stirring and allowed to react for 1 h at 50 °C. The reaction was stopped by diluting the resulting product with warm (40 °C) PBS and dialyzed against water for approximately 1 week. After lyophilization, codfish mGelMA was stored at 4 °C. (**B**) Scheme of the codfish gelatin reaction with MA to generate codfish mGelMA.

Gelatin methacrylation was performed following Nichol et al.’s [49] and Loessner et al.’s [50] studies as presented in Figure 1. Briefly, codfish gelatin was dissolved in PBS at 60 °C to produce a 10% (*w*/*v*) solution. After complete dissolution, methacrylic anhydride (MA) was added very slowly to the gelatin solution under vigorous stirring until an MA concentration of 20% (*v*/*v*) was reached. After 1 h of reaction at 50 °C, the reaction was stopped by diluting 5× with a PBS solution previously heated (40 °C). Excess unreacted MA and other low molecular weight components were removed by pouring the solution into a dialysis tubing membrane against distilled water at 40 °C until the solution appeared translucid and odorless. After dialysis, the pHs of mGelMA solutions were adjusted to pH = 7.4 and sterilized by filtration with 0.22 µm pore size filters and lyophilized for one week.

### 2.3. ^1^H-Nucleic Magnetic Resonance (^1^H-NMR) of mGelMA

The degree of methacrylation of mGelMA was quantified by ^1^H-NMR spectroscopy. Codfish gelatin and respective mGelMA were previously dissolved in deuterium oxide (D_2_O) at a concentration of 30 mg/mL at 40 °C. The ^1^H-NMR spectra were collected at 25 °C, recorded on a Bruker Avance III (Bruker, Germany) operating at a resonance frequency of 400 Hz. Spectra analysis was performed using Mestre Nova software (v12.0.0-20080). The spectra were normalized to the signal of phenylalanine (6.9–7.5 ppm), which represents the gelatin concentration. Then, the lysine methylene (2.8–2.95 ppm) signal was integrated to obtain the corresponding areas. The degree of functionalization of mGelMA (*DoF*) was calculated using the following formula and presented as a percentage (%) of the lysine ε-amino groups substituted with methacryloyl groups [51].

(1) $$DoF\;\left(\%\right)=\left(1-\left(\frac{Peak\;area\;lysine\;methylene\;of\;mGelMA}{Peak\;area\;lysine\;methylene\;of\;non-modified\;gelatine}\right)\right)\times 100$$

### 2.4. Production of mGelMA Hydrogels

To produce a mold for the mGelMA hydrogels, polydimethylsiloxane (PDMS) prepolymer–catalyst mixtures were poured into Petri dishes and cured at 37 °C for 24 h. The resulting PDMS films were peeled off from the Petri dishes, and holes with specific dimensions were punched: 0.6 cm of diameter and around 0.5 mm of thickness to produce mGelMA discs for transparency tests; 1 cm of diameter and around 0.5 mm of thickness to produce mGelMA discs for the remaining tests. Solutions of 10% and 15% (*w*/*v*) with 0, 0.5, and 5 mg/mL ascorbic acid were prepared from codfish mGelMA and mixed with a 0.1% (*w*/*v*) Irgacure 2959 photoinitiator in PBS under stirring. To prepare the discs, 30 µL (for transparency tests) and 80 µL (for the remaining assays or otherwise mentioned) of the resulting solutions were poured into the PDMS molds and exposed to ultraviolet light ((366 nm, UV lamp Triwood 6/36; Bresciani srl., Milano, Italy) 0.120 J/cm^2^) for 2 min. The hydrogels produced were further designed as 0 AA, 0.5 AA, and 5 AA corresponding to 0, 0.5, and 5 mg/mL of ascorbic acid, respectively.

### 2.5. Characterization of mGelMA Hydrogels

#### 2.5.1. Chemical Analysis

Fourier transform infrared spectroscopy with attenuated total reflectance (ATR-FTIR) analysis was performed at room temperature using a FTIR Shimadzu-IR Prestige 21 spectrometer, in a spectral region of 4000 to 400 cm^−1^ with a resolution of 4 cm^−1^, using the average of 32 individual scans in absorbance mode to produce each spectrum.

#### 2.5.2. Equilibrium Water Content (EWC)

To determine the equilibrium water content, mGelMA discs were soaked in PBS at 37 °C for 24 h. The hydrogels were then gently blotted with filter paper to remove the superficial water and weighed (*Ww*). Gelatin methacryloyl hydrogels were lyophilized for two days, and the weight was recorded again (*Wd*). The *EWC* (% (*w/w*)) was calculated as follows:

(2) $$EWC\;\left(\%\right)=\frac{Ww-Wd}{Ww}\times 100$$

The reported values were represented as the average of at least five measurements.

#### 2.5.3. Degradation in PBS

For the degradation assay, mGelMA hydrogels were lyophilized and weighed (*Wi*). The hydrogels were then incubated in PBS, at 37 °C with constant shaking (120 rpm), for a specific period of time (1, 7, 14, and 21 days). At each timepoint, the hydrogels were washed with distilled water, lyophilized, and weighed (*Wf*). The weight loss of the hydrogels was calculated using the following equation:

(3) $$Weight\;loss\;\left(\%\right)=\frac{Wi-Wf}{Wi}\times 100$$

At least six hydrogels per condition were used for measurements.

#### 2.5.4. Enzymatic Degradation

To determine stability in an enzymatic environment, mGelMA hydrogels were lyophilized, weighed (*Wi*), equilibrated in PBS for 24 h, and then incubated in PBS with 2 µg/mL collagenase A at 37 °C up to 72 h. After 1, 2, 4, 6, 24, 48, and 72 h of incubation, the mGelMA hydrogels were rinsed in distilled water, lyophilized, and weighed (*Wf*). Weight loss was calculated as described previously. A minimum of six replicates were used for each hydrogel condition.

#### 2.5.5. Mechanical Tests

The mechanical properties were evaluated under both uniaxial tensile and compressive modes using a universal mechanical testing machine (5543, Instron) equipped with a 50 N load cell. For the tensile tests, hydrogel specimens of each condition were produced using specific PDMS molds with rectangular shape form with total length of 40 mm, width of 5 mm, and an average thickness of 1.20 mm before being immersed in PBS for 24 h. The samples were then clamped onto a sandpaper-coated metal jaw at a gauge length of 10 mm. For the compressive tests, cylindrical mGelMA hydrogel specimens with a diameter of 7 mm and average thickness of 3.5 mm were also prepared in a distinct silicon mold before being immersed in PBS for 24 h. A crosshead speed of 2 mm/min was applied for both tests. At least five specimens per condition were tested. The maximum tensile strength and elongation at break, and the compressive strength were recorded. The elastic modulus under the tensile or compression mode was then calculated from the stress–strain curve as the slope of the initial linear portion of the curve.

#### 2.5.6. Transparency of mGelMA Hydrogels

The macroscopic appearance and transparency of the gels were assessed by placing them over printed text. The light transmittance of the hydrogels was assessed on discs with 0.6 cm diameter and 0.5 mm thickness (*n* = 3). Hydrogels were produced and incubated in PBS at 37 °C and at specific time points (1, 7, 14, and 21 days) and transferred to a 96-well plate with 100 µL of PBS. Wells containing only PBS were used as blanks. The transmittance of hydrogels was measured using a microplate reader (Synergy HT, Bio-TEK, Winooski, VT, USA) under wavelength ranging between 380 and 800 nm and calculated by using the follow equation where the transmittance (*T*) of mGelMA hydrogels corresponded to the absorbance (*A*) as shown below:

(4) $$T=10^{-A}$$

#### 2.5.7. Release Profile of Ascorbic Acid

To confirm the presence and evaluate the release behavior of AA, mGelMA hydrogels with different concentrations of AA were produced as described above, immersed in 4 mL of PBS and placed at 37 °C under orbital shaking (120 rpm). At different timepoints (0.5, 1, 2, 4, 8, 24, 48, and 72 h and 7, 14, and 21 days), 1 mL of the solution was withdrawn, transferred into amber vials, and immediately replaced with an equal volume of fresh buffer to maintain the sink condition. The detection of AA was performed by using HPLC-UV method detection, at 265 nm [52]. The analyses were carried out on Waters Alliance HPLC system with Waters 2489 UV/Visible Detector (Waters, Milford, MA, USA). Chromatography was performed using an isocratic elution, and the mobile phase consisted of 2.5% ethanol in 25 mmol/L sodium dihydrogen phosphate (*v*/*v*) (pH 4.7), on a Zorbax SB-C18 column (4.6 × 250 mm^−5^ µM) at 30 °C. The injection volume was 10 µL and the flow rate was set at 0.5 mL/min. The amount of AA was quantified from the corresponding peak area by interpolation using the calibration curve, previously set using AA standard solutions. The cumulative release rate was calculated using the following equation:

(5) $$Cumulative\;release\;\left(\%\right)=\frac{V_{0}C_{n}+VC_{n-1}}{m}\times 100$$

where *C_n_* and *C*_*n*−1_ are the concentration of AA on sampling for *n* times and *n* − 1 times, respectively, *V*_0_ is the initial volume of the release solution, *V* is the sampling volume, and *m* is the mass of AA loaded in the hydrogel.

### 2.6. Production of Cell-Loaded mGelMA Hydrogels

#### 2.6.1. Cell Culture

In vitro studies were performed using human keratocytes (HK), cultured following the company’s instructions. Briefly, cells were maintained in precoated poly-lysine T75 flasks in complete fibroblast medium (fibroblast medium containing 2% fetal bovine serum (FBS), 1% fibroblast growth supplement (FGS), and 1% penicillin/streptomycin solution), at 37 °C in a humidified atmosphere containing 5% CO_2_. Medium was exchanged every 2–3 days. All experiments were performed in HK cells at cumulative population doublings between 4 and 8.

#### 2.6.2. Cell Encapsulation and Hydrogel Preparation

To produce cell-loaded hydrogels, mGelMA was dissolved in complete fibroblast medium and the resulting solutions were handled as previously mentioned in Section 2.4. HK cells were dissociated using TrypLE™ Express enzyme and counted in a hemocytometer. After centrifugation, the cell pellet was gently mixed with mGelMA solution to prepare a 1 × 10^6^ cells/mL cell suspension as precursor solution, which was immediately transferred to previously sterilized PDMS molds (80 µL/hydrogel) and exposed to UV light as in Section 2.4. Crosslinked cell-laden hydrogels were carefully removed from the PDMS molds, washed in complete fibroblast medium, transferred to 24-well plates, and then incubated with the same medium at 37 °C in a humidified atmosphere containing 5% CO_2_. The medium was changed each 2–3 days.

### 2.7. Performance of the Encapsulated Cells

#### 2.7.1. Cell Viability

##### Live–Dead Staining

To assess cell viability, a Live–Dead staining was performed. Cells were labeled with calcein AM (live cells in green) and propidium iodide (PI, dead cells in red) on day 1 and 3. Briefly, cell medium was removed and replaced by a mixture of calcein AM (CA, 4 µg/mL) and PI (1 µg/mL) diluted in a culture medium solution. After 30 min incubation at 37 °C in a humidified atmosphere containing 5% CO_2_, hydrogels were immediately examined under Confocal Laser Scanning Microscopy (TCS SP8, Leica, Wetzlar, Germany). The acquisition of hydrogels without cells was performed to detect nonspecific staining. Cell viability (%) was calculated by counting the live and dead cells on 3D structures generated by LAS X Image Analysis (3D) software and using the following equation:

(6) $$Cell\;viability\;\left(\%\right)=\frac{Live\;cells\;\left(green\right)}{Total\;number\;of\;cells\;\left(green+red\right)}\times 100$$

The quantification results were presented as mean ± SD of 2 independent experiments with 2 replicates and 3 images per replicate.

##### MTS Assay

The metabolic activity was assessed by the [3-(4,5-dimethylthiazol-2-yl)-5-(3-carboxymethonyphenol)-2-(4-sulfophenyl)-2H-tetrazolium, inner salt] (MTS) assay (CellTiter 96 AQueous One Solution, Promega, Madison, WI, USA), which is based on the reduction of tetrazolium compound by viable cells to generate a colored formazan dye. At the endpoints of days 1 and 3, the culture medium was removed, and hydrogels were washed with sterile DPBS. The cell-laden hydrogels were incubated with a mixture of culture medium (DMEM low glucose without FBS and phenol red) and MTS reagent (5:1 ratio) for 3 h, at 37 °C in a humidified atmosphere containing 5% CO_2_. After that, the hydrogels were placed in an orbital shaker for an additional 30 min to allow the diffusion of the reduced agent into the medium. Then, 100 μL of MTS reaction medium was transferred to a 96-well plate in duplicate and the absorbance was measured at 490 nm in a microplate reader (Synergy HT, Bio-TEK). The results were expressed as a percentage relative to the control (hydrogels without ascorbic acid). Values are shown as mean ± SD (*n* = 4).

##### Immunofluorescence Staining

Immunofluorescence staining was performed to study the expression of specific antibodies. Samples were fixed with 4% (*v*/*v*) paraformaldehyde for 30 min at room temperature (RT), washed with PBS, and incubated with 1% Triton X-100 in PBS for 20 min to enhance cell membrane permeability. After washing, nonspecific interactions were blocked by incubation with 3% bovine serum albumin (BSA) in PBS for 30 min. Primary antibodies to detect collagen I (COLI) (1:200 *v/v* in 1% BSA + 0.2% Triton X-100), collagen V (COL V) (1:25 *v/v* in 1% BSA + 0.2% Triton X-100), decorin (1:500 *v/v* in 1% BSA + 0.2% Triton X-100), and lumican (1:200 *v/v* in 0.1% BSA + 0.2% Triton X-100) were prepared. After blocking, samples were incubated with previously mentioned primary antibodies at 4 °C overnight. Next day, the hydrogels were washed with PBS and incubated with the respective secondary antibody (1:500, anti-rabbit or anti-goat) for 1 h at RT. Cell nuclei were stained with amidino-2-phenylindole (DAPI, 2 µg/mL). At the end, samples were washed with PBS and imaged using Confocal Laser Scanning Microscopy (TCS SP8, Leica, Wetzlar, Germany) or kept in PBS at 4 °C in the dark until analysis. Secondary antibody-treated mGelMA hydrogels were used as negative controls. The images were taken using a 20× immersion objective.

### 2.8. Statistical Analysis

Statistical analysis was performed using SPSS software (IBM SPSS Statistics version 27, Armonk, NY, USA), and graphical drawing was performed using GraphPad Prism (version 8.0) (San Diego, CA, USA). Data normality and homogeneity of variances were evaluated using the Shapiro–Wilk and Levene tests, respectively. If the data followed a normal distribution, one-way ANOVA followed by Fisher’s LSD test was used. If the data failed to pass those tests, the Kruskal–Wallis test followed by Fisher’s LSD test was performed. For the two-group comparison, an unpaired *t*-test (Mann–Whitney U-test) for nonnormally distributed variables was performed. Statistical significance was defined as *p* ≤ 0.05. Data were expressed as means ± standard deviation of experiments with at least two independent experiments.

## 3. Results

### 3.1. Gelatin Methacrylation

**Figure 2:**
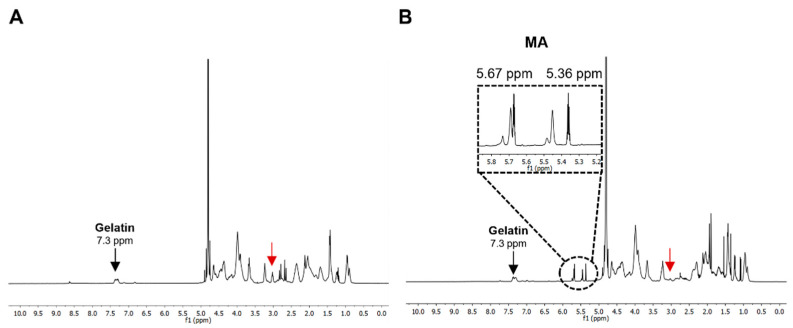
^1^H-NMR spectra of codfish gelatin (**A**) and codfish gelatin methacryloyl (**B**). The red arrow represents the lysine methylene peak.

The success of the methacrylation reaction is determined by measuring the degree of functionalization (DoF), and it is defined as the percentage of amine groups (lysine, hydroxylysine) of gelatin that were functionalized with methacrylic anhydride (MA), as described in Section 2.3. Quantitative ^1^H-NMR analysis was used to determine the extent of the reaction. Figure 2 shows the spectra relative to the unmodified codfish gelatin (Figure 2a) and the spectra of codfish gelatin confirming the reaction with MA groups (Figure 2b), as new peaks could be observed at δ = 5.36 ppm and δ = 5.67 ppm, specific to the methacryloyl group. In addition, the decrease in the lysine methylene relative peak, assigned by the red arrow, is an indication of the modification of the lysine groups. The quantitative results showed that with the protocol used, mGelMA had approximately 75% DoF.

### 3.2. Production of mGelMA Hydrogels and Characterization

**Figure 3:**
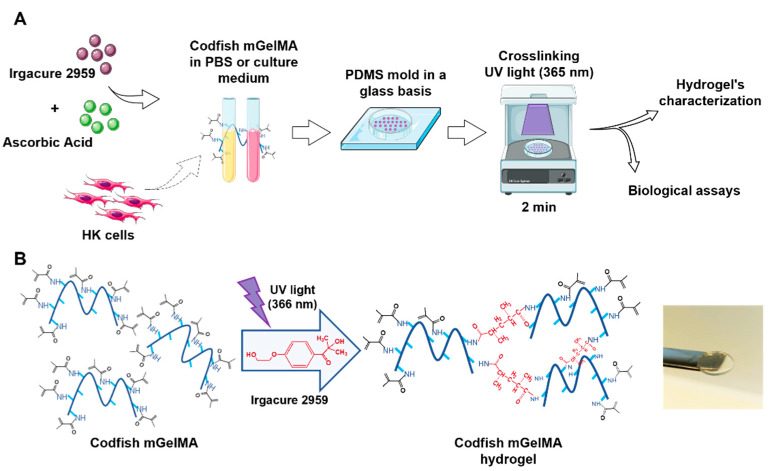
Summary of codfish mGelMA hydrogel assembly. (**A**) Schematic representation of hydrogel production with or without HK cells. (**B**) Representative scheme of the reaction between codfish mGelMA polymer and the photoinitiator to obtain crosslinked hydrogels.

The preparation of the mGelMA hydrogels for chemical and physical characterization, as well as for biological assays, is summarized in Figure 3. Codfish mGelMA and the photoinitiator (Irgacure 2959) were dissolved in the presence or absence of ascorbic acid, and the resulting solution was poured into a PDMS mold and crosslinked under UV light for 2 min. Cohesive and structurally stable hydrogels were obtained using both 10% and 15% mGelMA solutions.

#### 3.2.1. ATR-FTIR Analysis

**Figure 4:**
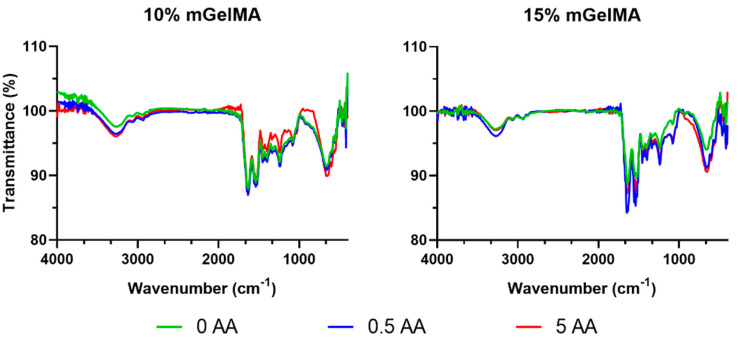
ATR-FTIR spectra of 10% and 15% mGelMA hydrogels with and without ascorbic acid (AA).

The spectra of mGelMA hydrogels with and without AA were investigated by ATR-FTIR spectroscopy as shown in Figure 4. First, it was observed that for both 10% and 15% mGelMA, the characteristic peaks of gelatin molecules were present. The peak signal of amide A (≈3275 cm^−1^) was attributed to the O–H and N–H stretching vibrations, amide B (≈3064 cm^−1^) was assigned to the stretching vibration of C–H groups, the amide I (≈1641 cm^−1^) was relative to C=O stretching, the amide II (≈1538 cm^−1^) was assigned to N–H bending coupled to C–H stretching, and amide III (≈1237 cm^−1^) was associated with C–N stretching and N–H bending. Regarding the presence of AA, due to the low relative amount of AA incorporated in the constructs, the FTIR technique could not detect its presence as it was not possible to distinguish characteristic peaks that were overlapping with the ones from mGelMA. Thus, the HPLC-UV method was used both to confirm the incorporation of AA in the hydrogels and quantify its release.

#### 3.2.2. Equilibrium Water Content of mGelMA Hydrogels

**Figure 5:**
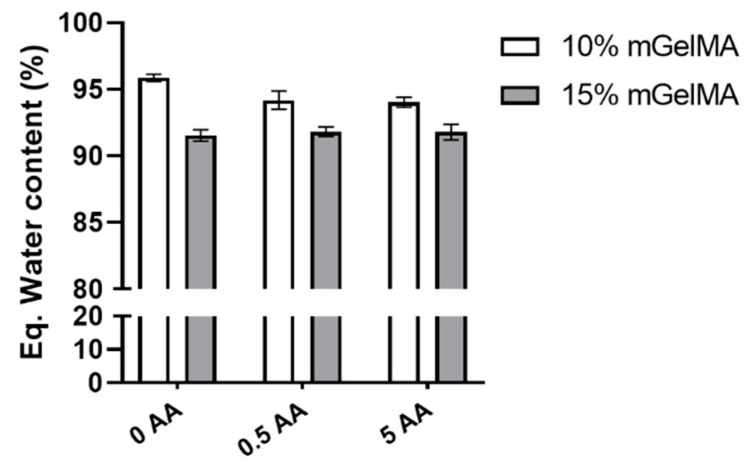
Water content variation at equilibrium state of 10% and 15% mGelMA hydrogels with different ascorbic acid concentrations. The values shown are means ± SD (*n* = 9).

The equilibrium water content of the mGelMA hydrogels was evaluated and the results are presented in Figure 5. As observed, all conditions retained high levels of water (above 90% water content). No variation was observed between mGelMA hydrogels with and without AA. It was also noticed that no statistical significance was found between the conditions of 10% and 15% mGelMA. However, a tendency for lower water content was observed for hydrogels with 15% mGelMA (approximately 95% water content for 10% mGelMA and 92% for 15% mGelMA).

#### 3.2.3. Stability of mGelMA Hydrogels and Release of AA

**Figure 6:**
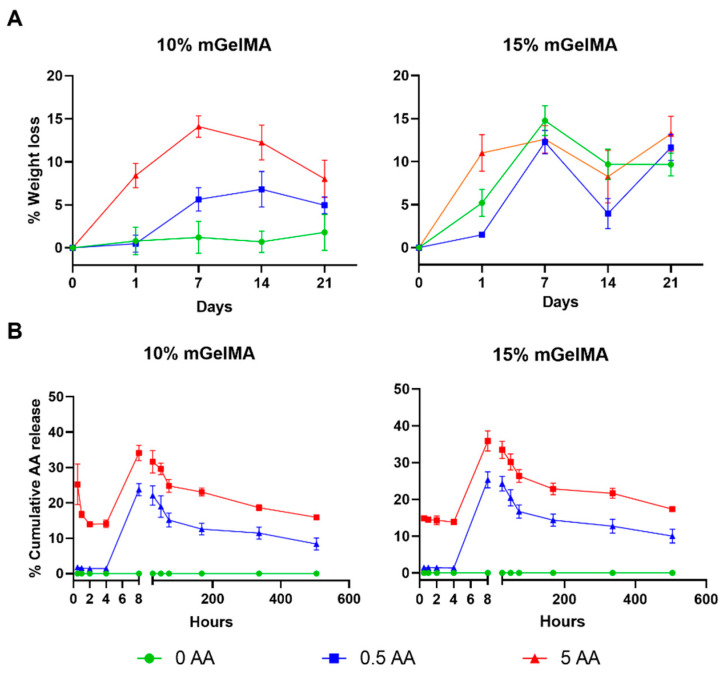
Degradation of mGelMA hydrogels in PBS and ascorbic acid (AA) release. (**A**) Degradation profile of mGelMA hydrogels with different AA concentrations (0, 0.5, or 5 mg/mL) in 21 days in PBS at 37 °C. (**B**) Ascorbic acid cumulative release profile during 21 days at 37 °C. Values are shown as mean ± SD (*n* = 6).

The stability of the mGelMA hydrogels was assessed by PBS and enzymatic degradation. Simultaneously with PBS degradation, the release of ascorbic acid into the medium was measured. The hydrogels were immersed in PBS for 21 days, and at the end of each time point, the weight loss was recorded as shown in Figure 6a. In the case of the 10% mGelMA hydrogels, the condition without ascorbic acid (0 AA) showed almost no degradation over time. When AA was added to the hydrogel, an increase in the weight loss rate was observed, with the hydrogels containing 0.5 AA presenting a maximum weight loss of 6.8 ± 2.1% on day 14 of incubation. With higher concentrations of AA, the process started at earlier time points, reaching a maximum weight loss of 14.1 ± 1.3% at day 7. Regarding 15% mGelMA hydrogels, the degradation process was detected at earlier timepoints for all conditions, with a non-monotonous variation over time. To highlight that, on day 1 of incubation, the condition that presented the highest weight loss was the one encompassing 5 AA (weight loss of 11 ± 2.1%).

Simultaneously to the degradation assay in PBS, the detection and release of AA from the hydrogels to the incubation medium was measured using quantitative HPLC (Figure 6b). It was observed that the AA was successfully incorporated into the hydrogels. The results demonstrated that the AA release pattern was similar for both hydrogels (10 and 15% mGelMA). For 10% mGelMA hydrogels, the 5 AA condition showed a fast release of AA (25.2 ± 5.7%) at earlier time points (30 min of incubation), whereas in the 0.5 AA condition, the detected release was very low (1.8 ± 0.1%). For both the 0.5 AA and 5 AA conditions, the maximum release was observed at 8 h of incubation with 23.8 ± 1.7% and 34.1 ± 2.2%, respectively. For the hydrogels produced from 15% mGelMA solutions, a similar behavior was observed. At 30 min of incubation, the 5 AA condition showed a rapid release of AA (14.8 ± 0.4%) when compared with the 0.5 AA condition (1.4 ± 0.02%). Moreover, a maximum release was detected at 8 h of incubation with 25.3 ± 2.2% and 35.9 ± 2.7%, for 0.5 AA and 5 AA, respectively.

**Figure 7:**
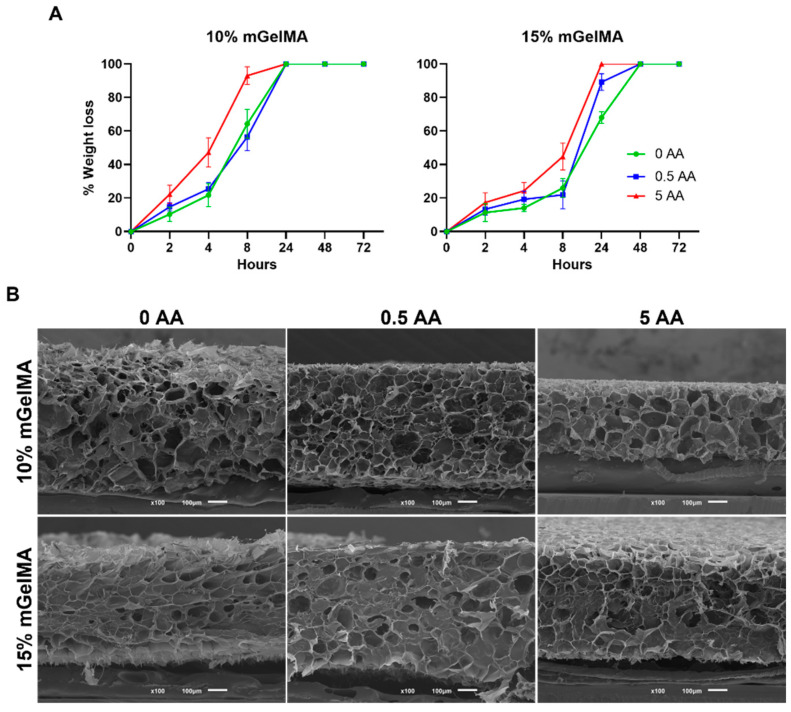
In vitro enzymatic degradation profile and resultant appearance of mGelMA hydrogels. (**A**) Weight loss of the mGelMA hydrogels as result of enzymatic degradation promoted by incubation in the collagenase-containing PBS at 37 °C. (**B**) SEM micrographs of cross-section of mGelMA hydrogels after 8 h of incubation with collagenase type II. Scale bars are 100 µm.

To closely mimic the in vivo degradation conditions, the integrity of the mGelMA hydrogels was evaluated using collagenase to promote the enzymatic degradation of the hydrogel (Figure 7). The results from Figure 7a show that, at earlier time points, 15% mGelMA hydrogels were more stable in terms of enzymatic activity, with about 20% of weight loss for 0 and 0.5 AA and 40% of weight loss for 5 AA at 8 h of incubation. After 48 h of incubation, all hydrogels were fully degraded. Under the conditions with 10% mGelMA, it was possible to observe that the hydrogels were completely digested after 24 h of incubation, more rapidly than their 15% mGelMA counterparts. It is also important to highlight that the presence of AA in the hydrogel affects the degradation rate. Increasing the concentration of AA led to faster degradation of the hydrogels. Figure 7b shows a micrograph of the mGelMA hydrogel after 8 h of incubation with collagenase type II. It is possible to see the existence of large pores and sheet-like structures on the hydrogels. Hydrogels of 10% mGelMA and 5 AA were thinner, which is in agreement with previous results.

#### 3.2.4. Mechanical Properties of mGelMA Hydrogels

**Figure 8:**
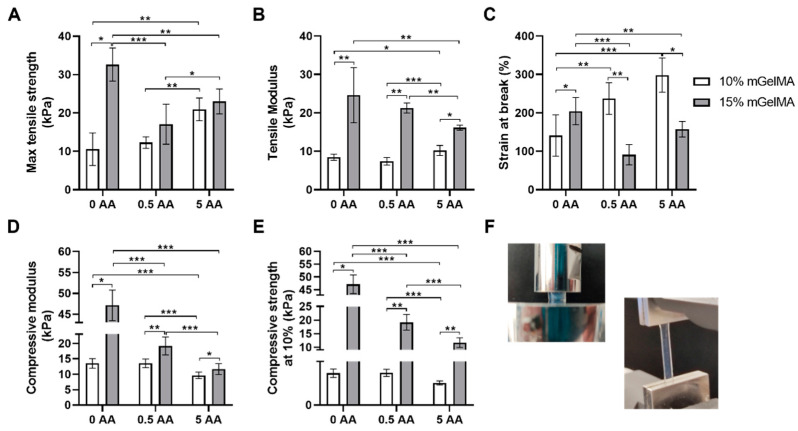
Mechanical properties of mGelMA hydrogels. (**A**) Maximum tensile strength (kPa), (**B**) tensile Young’s modulus (kPa), (**C**) maximum strain at break (%), (**D**) compressive Young’s modulus (kPa), (**E**) compressive strength determined at 10% of strain (kPa), (**F**) representative image of the setup used. The values shown are means ± SD (*n* = 5). * Denotes statistically significant difference (*: *p* ≤ 0.05; **: *p* ≤ 0.005; ***: *p* ≤ 0.0005).

The mechanical properties of the mGelMA hydrogels under uniaxial tensile and compression loads were evaluated, and the obtained results are depicted in Figure 8. Regarding the different concentrations of mGelMA tested, the tensile properties revealed that the maximum strength and stiffness of the hydrogels tended to increase with the increase in mGelMA concentration. The tensile strength of 15% mGelMA increased tendency, with statistical significance for 0 AA hydrogels when compared with 10% mGelMA hydrogels (Figure 8a). The strength values were around 10.5–20.9 kPa for 10% mGelMA, and 17.1–32.6 kPa for 15% mGelMA. In addition, the tensile modulus exhibited the same behavior, with 15% mGelMA hydrogels showing higher modulus than 10% mGelMA, independently of the AA concentration (Figure 8b). The values for tensile modulus were between 7.4 and 10.2 kPa for 10% mGelMA, and 16.1 and 24.6 kPa for 15% mGelMA. In contrast, the maximum strain at break of the 15% mGelMA hydrogel was higher than that of the 10% mGelMA (only for hydrogels with 0 AA). In the presence of AA, the strain at break of 10% mGelMA was greater than that of 15% mGelMA (Figure 8c). Different behaviors were observed when AA was added to the hydrogels. In 10% mGelMA hydrogels, the incorporation of AA resulted in higher mechanical properties, increasing with AA concentration (Figure 8a–c), whereas in 15% mGelMA hydrogels, the addition of AA originated an inverse behavior, resulting in a significant decrease in the tensile properties.

Despite this observation, all the produced hydrogels presented good dimensional stability and ductile fracture behavior, revealing promising properties for the proposed function.

In terms of mechanical behavior under a compressive load, our results demonstrated that higher mGelMA concentrations resulted in a higher compressive modulus (Figure 8d), with the addition of AA having a deleterious effect on the compressive properties. For 10% mGelMA, the higher compressive modulus was observed for 0 and 0.5 AA (13.5 ± 1.5 kPa and 13.5 ± 1.4 kPa, respectively) conditions, and the lowest was detected for the 5 AA (9.6 ± 1.1 kPa) condition. Regarding the 15% mGelMA hydrogels, a higher compressive modulus was observed for the 0 AA (47.1 ± 3.7 kPa) condition, and the lowest was detected for the 5 AA (11.7 ± 1.8 kPa) condition. Considering an initial compression of 10% of strain, the obtained strength values were around 1.0–1.4 kPa for 10% mGelMA, and around 11.7–47.1 kPa for 15% mGelMA (Figure 8e). An illustrative image of the setup used during the tests is presented in Figure 8f. Considering the proposed strategy, it was possible to observe that the mechanical properties under compressive load could be modulated by the mGelMA content in the hydrogels to achieve the required properties.

#### 3.2.5. Optical Properties of mGelMA Hydrogels

**Figure 9:**
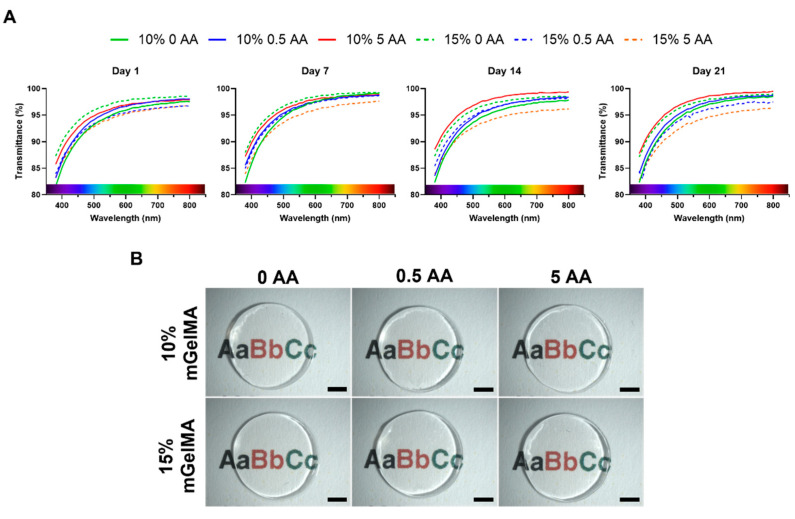
Optical properties of mGelMA hydrogels over time. (**A**) Spectral transmittance of mGelMA hydrogels at day 1, 7, 14, and 21. Values shown are means (*n* = 8). (**B**). Transparency of the hydrogels illustrated by photography over text. Scale bars are 2000 µm.

The optical properties of the mGelMA hydrogels were evaluated by assessing their light transmittance and transparency within the visible spectrum (380–800 nm), and the results are shown in Figure 9. There were no significant differences between the light transmittances of the different mGelMA hydrogels (Figure 9a). Furthermore, the transmittance of the hydrogels remained almost constant over time, with only a slight variation between hydrogel conditions on day 14. All the hydrogels exhibited a transmittance above 80%.

### 3.3. Encapsulation of Human Keratocytes

#### 3.3.1. Cell Viability

**Figure 10:**
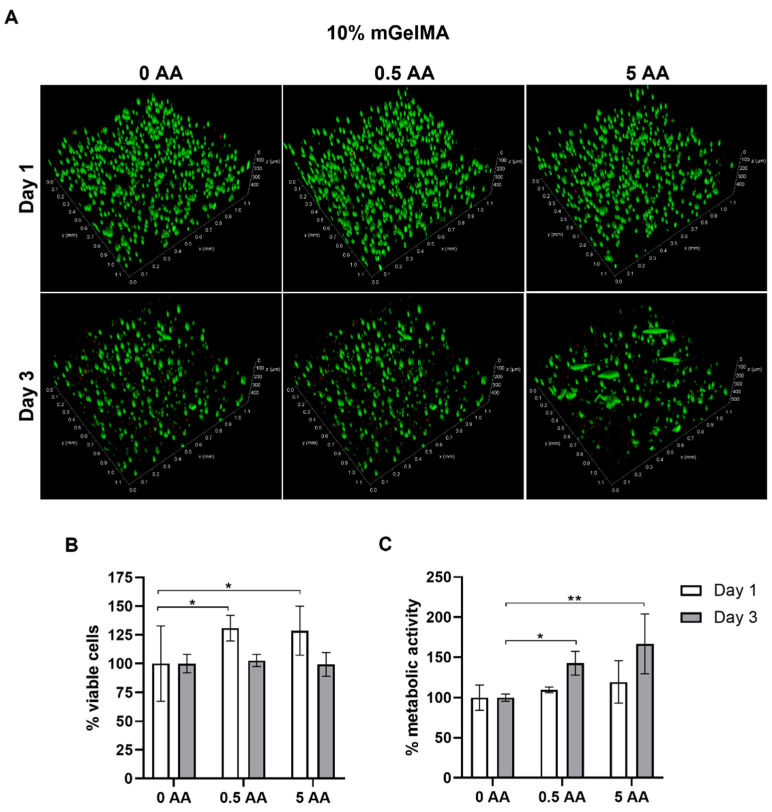
Viability of human keratocytes (HK) loaded in 10% mGelMA hydrogels. (**A**) Live–dead images of mGelMA hydrogels with 0, 0.5, and 5 AA on days 1 and 3. Viable cells were stained in green color (calcein AM) and dead cells were stained in red (PI). (**B**) Quantitative analysis of fluorescence of viable cells (%) over 3 days of culture. (**C**) Metabolic activity of HK cells at days 1 and 3 as determined by MTS assay. Results are expressed as percentage relative to the control (hydrogel without AA). Values are shown as mean ± SD (*n* = 4). * Denotes statistically significant difference (*: *p* ≤ 0.05; **: *p* ≤ 0.005).

**Figure 11:**
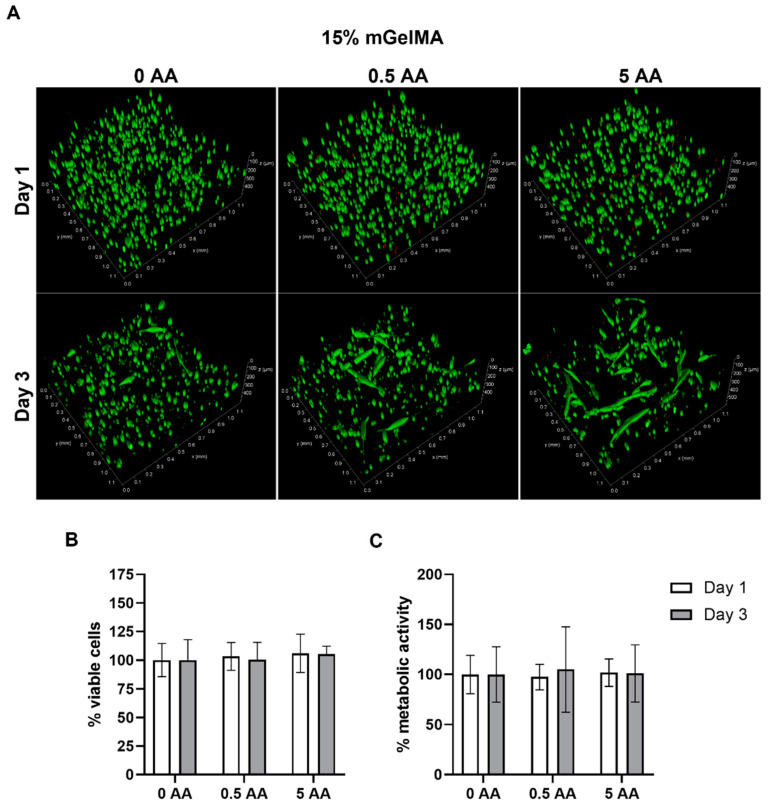
Viability of human keratocytes (HK) loaded in 15% mGelMA hydrogels. (**A**) Live–dead images of mGelMA hydrogels with 0, 0.5, and 5 AA on days 1 and 3. Viable cells were stained in green color (calcein AM) and dead cells were stained in red (PI). (**B**) Quantitative analysis of fluorescence of viable cells (%) over 3 days of culture. (**C**) Metabolic activity of HK cells at days 1 and 3 as determined by MTS assay. Results are expressed as percentage relative to the control (hydrogel without ascorbic acid). Values are shown as mean ± SD (*n* = 4).

Cell viability in the 10% mGelMA and 15% mGelMA hydrogels was evaluated by live–dead and MTS assays, as shown in Figure 10 and Figure 11, respectively. The live–dead qualitative results showed a successful distribution of the incorporated living cells in all mGelMA hydrogel formulations, as observed by the green signal assignment. For the 10% mGelMA hydrogels (Figure 10), the live–dead results showed that, on day 1, the HK cells presented a round shape and were spread over the hydrogel. This was verified for conditions with and without AA. On day 3, the number of cells was lower for all tested conditions, as indicated by the lowest green signal intensity. However, the number of elongated (fibroblast-like) cells was higher, particularly in the condition with 5 AA (Figure 10a). These results were corroborated by the quantification of viable cells, which showed that on day 1, the presence of AA was beneficial to HK (*p* ≤ 0.05) (Figure 10b). In contrast, there were no significant differences on day 3 among all the tested conditions. The evaluation of metabolic activity by MTS demonstrated that on day 3, HK cells were more metabolically active on hydrogels loaded with 5 AA (*p* ≤ 0.005) (Figure 10c).

For the 15% mGelMA hydrogels (Figure 11), live–dead confocal images showed that, on day 1, cells were also mostly round and spread over the hydrogel, while on day 3, they presented a more elongated shape, particularly on the 5 AA condition (Figure 11a). Live–dead fluorescence quantification (Figure 11b) showed no differences in the number of viable cells between the conditions with or without AA. In addition, no differences in metabolic activity were observed under either condition (Figure 11c).

#### 3.3.2. Immunofluorescence

**Figure 12:**
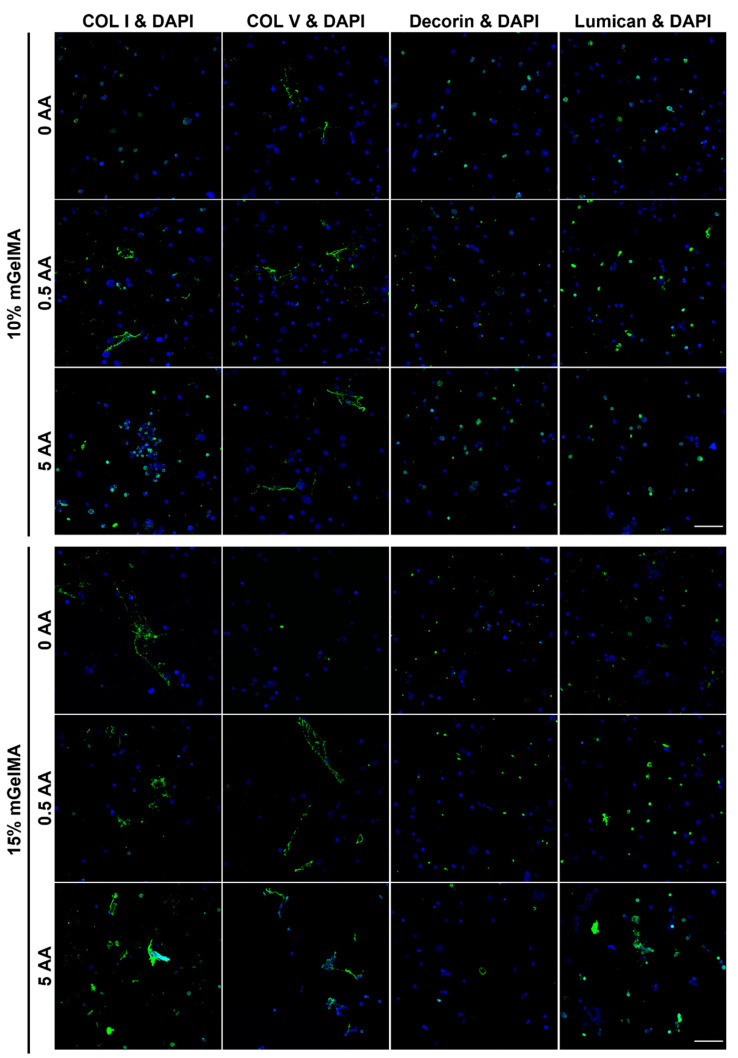
Immunofluorescence micrograph of mGelMA hydrogels at day 21 with collagen type I (COL I), collagen type V (COL V), decorin, and lumican, stained in green. Nuclei were visualized by staining with DAPI (blue). Scale bar are 100 µM.

HK corneal ECM proteins, COL I and V, as well as proteoglycans, decorin and lumican, expression was evaluated by immunofluorescence staining, as shown in Figure 12. The expression of COL I and V, decorin, and lumican in HK cells was observed to be dispersed over the hydrogels, although with different spatial distributions. The expression of COL I and V seemed to be more diffused, namely, for the hydrogel containing 10% mGelMA/0.5 AA, 15% mGelMA/0.5, and 5 AA. In contrast, the expression of proteoglycans seemed to be localized near the cells with lower dispersion. No nonspecific binging was detected in mGelMA hydrogels stained only with a secondary antibody (Supplementary Figure S1).

## 4. Discussion

The ultimate goal of TE is the production of a bioartificial construct that mimics the host tissue characteristics and provides an ideal environment for effective integration and repair. An endless set of materials and different production strategies can be used to achieve the desired architecture and biological cues. The use of collagenous marine-derived materials for corneal regeneration has been limited to a few reports, in which decellularized and decalcified fish scales were used as corneal substitutes [42]. Studies with this new fish scaffold showed good results, envisioning the forwarding to human clinical trials [43]. These results demonstrate the promising applicability of fish-derived products for corneal regeneration. Similarly, the use of methacrylated gelatin as a strategy for the fabrication of hydrogels has demonstrated good results both in vitro and in vivo, emerging as an alternative to corneal stromal regeneration [53,54,55]. Therefore, the present study aimed to evaluate the capacity of gelatin extracted from codfish skins to be functionalized with a photo-responsive methacryloyl (MA) group and used to produce hydrogels for corneal stromal applications. Furthermore, ascorbic acid (AA) was added to the system to enhance the production of collagen by cells, as well as the potential antioxidant protection [56]. This was the first time that marine-derived GelMA (mGelMA) was used to fabricate scaffolds loaded with AA for corneal stromal applications. First, marine gelatin, previously extracted from by-products (skins) of the codfish industry, was successfully methacrylated, as observed by the detection of the NMR signal, characteristic of MA specific groups, together with a decrease in lysine methylene peaks. These findings are in agreement with those described in previous reports [51,57] on methacrylation processes. Subsequently, 10% and 15% (*w*/*v*) mGelMA solutions with different concentrations of AA were jellified by photocrosslinking, with the FTIR spectroscopic analysis of the resulting hydrogels confirming the presence of gelatin characteristic bands, as also reported by Fonseca et al. [58].

The ability of a hydrogel to retain water is a crucial property. It has a strong impact on other properties such as the rearrangement of the network structure, mechanical features, molecule diffusion, transparency, as well as on the biological performance [59]. The equilibrium water content of the mGelMA hydrogels was slightly higher than that found by Bektas et al. [53] and Vigata et al. [60] also working with GelMA hydrogels. Although no statistical difference was observed between the tested conditions, it was possible to observe a tendency for higher mGelMA concentrations to retain less water, as reported as well by Vigata et al. [60]. This can be explained by the direct relationship between the hydrogel network density and the capacity to retain water [61]. With an increase in mGelMA concentration, the number of free-mGelMA molecules available to react with the photoinitiator increases and, consequently, an increase in crosslinking density occurs. Considering that the cornea is a tissue composed of approximately 78% water [62], the hydrogels produced herein are shown to be suitable for maintaining corneal hydration.

The stability of an implanted biomaterial is important for the successful regeneration process. Ideally, the biomaterial should degrade at the same rate as new tissue is formed [63]. The evaluation of the degradation rate of a biomaterial under physiological conditions provides insight into its expected behavior in vivo. In this work, hydrogel degradation was first evaluated in PBS for 21 days. It would be expected that more concentrated mGelMA hydrogels present lower degradation rates, as described by Bektas et al. [53]. However, the results showed that the 15% mGelMA hydrogels (without AA) presented a higher weight loss than the 10% mGelMA hydrogels (without AA) on day 7. The most probable reason for this variation may be the leaching out of the uncrosslinked polymer that did not react within the 2 min of UV exposure. However, the percentage of weight loss did not exceed 15%. When AA was added to the hydrogels, the percentage of weight loss was considerably higher (for both the 10% and 15% mGelMA hydrogels), especially when loaded with 5 AA. This could be due to the diffusion of AA from the hydrogel or the loss of uncrosslinked mGelMA. As previously mentioned, AA plays an important role in biological systems. On the one hand, it is an essential cofactor during collagen biosynthesis [46] and, on the other hand, it has antioxidant activity offering protection to cells and tissues from oxidative stress [45]. Therefore, supplementation with this molecule could be beneficial to the corneal stromal tissue by enhancing cellular performance. Therefore, it is important to analyze the release profile of AA. Ascorbic acid release was simultaneously evaluated with hydrogel degradation in PBS. At both mGelMA concentrations, hydrogels loaded with 5 AA showed a higher release rate than 0.5-AA-loaded hydrogels. This was also observed in a report by Luo et al. [48], where higher AA-loaded gelatin cryogels released more antioxidant molecules. For the condition loaded with 5 AA, a release within the first hour was observed, which may be explained by the fact that the hydrogels used for the experiment were in a freeze-dried state and were placed directly in PBS medium. Therefore, during the first 2 h, the outer surface of the hydrogels started to expand and swell, while the inner part was still dry, leading the AA present in the outer part of the hydrogels to immediately diffuse into the medium upon incubation in PBS. No differences were found in the release rates between the 10% and 15% mGelMA hydrogels. Nevertheless, a diffusion of AA over time was observed, indicating that it can be released from the hydrogel in a sustained way, being available not only for the encapsulated cells but also to the cells surrounding the hydrogels when implanted. Additionally, the degradation was evaluated under enzymatic action to better estimate the stability of the hydrogels in vivo. For the bacterial collagenase test, all hydrogels showed accelerated degradation, ranging from 1 h to 24 h for 10% mGelMA and from 1 h to 48 h for 15% mGelMA. The increase in mGelMA content has been shown to strengthen the constructs against collagenase activity, as higher concentrations of mGelMA resulted in a higher crosslinking density. With the addition of AA, the hydrogels became more unstable, and the degradation rate increased as the concentration of AA increased for both mGelMA concentrations. This correlation between the presence of AA and hydrogel stability was also observed in previous studies [48].

The evaluation of the mechanical properties of the hydrogels showed that the concentration of mGelMA had an impact on the mechanical performance [64]. It was observed that hydrogels with higher concentrations exhibited higher tensile and compressive properties. As already mentioned, the increase in mGelMA concentration increased the complexity of the polymeric network density, and consequently, the mechanical properties were improved, in agreement with the findings reported by others [53,65]. The addition of AA has also been shown to affect the mechanical properties of mGelMA hydrogels. The AA incorporation decreased the compressive properties of hydrogels produced with both mGelMA concentrations. Concerning the tensile properties, an opposite behavior was observed between the 10% and 15% mGelMA concentrations. Herein, 15% mGelMA hydrogels exhibited a decrease in tensile properties with increasing AA concentrations. In contrast, 10% mGelMA hydrogels presented an increase in tensile properties with the addition of AA. Somehow, the inclusion of AA to low-mGelMA-concentrated hydrogels might induce a mechanical reinforcement of the polymeric network. Luo et al. [48] demonstrated that incorporating AA into cryogels resulted in a higher pore size and porosity, which may result in a lower resistance to compressive forces. This may explain why the compressive mechanical properties decreased with the increase in AA concentrations. On the other hand, the higher pore size and porosity can result in a less dense and complex polymeric network. Thus, when exposed to constant tensile stress, this allows a progressive alignment of polymeric chains caused by interfibrillar slippage. This was particularly evident for the 10% mGelMA hydrogels, which are less dense, thus explaining the increase in tensile mechanical properties with the increase in AA concentration. In the literature, the values for native corneal mechanical properties are found to be 38 kPa for the compressive modulus [66], 3.8 MPa for tensile strength [67], and between 0.9 and 9 MPa for the tensile modulus [68]. Although the mechanical properties of the developed hydrogels did not completely fulfill the requirements of the native cornea, they still hold promising qualities for the promotion of corneal stromal regeneration. Nonetheless, if desired, these properties could be enhanced by the addition of small amounts of synthetic polymers suitable for ophthalmic purposes, such as poly(methyl methacrylate) (PMMA), poly(hydroxyethyl methacrylate) (pHEMA), and poly(lactic-co-glycolic acid) (PLGA) [69].

The transmittance of the mGelMA hydrogels was above 80% over 21 days of incubation. The observed variations between the different mGelMA concentrations and AA loadings were probably related to the degradation rate. The native cornea has a light transmittance of 85% at 500 nm [70]. The obtained results indicated that, regardless of the tested hydrogel, the transparency of mGelMA hydrogels was similar to that of the human cornea, suggesting that its clinical use would not impair vision during the initial stages of the regenerative process. Nevertheless, corneal regeneration is mostly dependent on cell behavior in the hydrogels, namely, regarding ECM remodeling. Although both the mGelMA concentrations and AA loading conditions were compatible with HK encapsulation, the results indicated that 10% mGelMA hydrogels were more suitable and beneficial for cellular sustainability. The lower viability results obtained with the higher mGelMA concentration may be related to the superior polymer network density, which increased the stiffness of hydrogels and caused greater stress for cells, resulting in cell death. In addition, higher mGelMA concentrations were reported to be related to less porous structures due to the increase in network density, thus hampering nutrient and gas diffusion, as well as cellular attachment and spreading [65]. The presence of AA in the hydrogels demonstrated a favorable effect on HK metabolic activity, as corroborated by previous reports [48]. The production of extracellular matrix by HK cells was evaluated by the expression of representative collagens and proteoglycans present in the corneal stroma. The positive expression of COL I and V, decorin, and lumican is indicative of the preserved phenotype of HK encapsulated cells [53,71]. Moreover, there was an indication of the beneficial role of AA in the expression of COL I and COL V, in agreement with the observations made by Guo et al. using a transwell culture of HK [56], with the 0.5-AA-loaded mGelMA hydrogels presenting a higher expression of these proteins important for the ECM remodeling. The results seemed promising regarding the regenerative potential of these hydrogels; however, future studies planning a follow-up on gene-related markers and the detection of other important ECM markers need to be addressed.

The mGelMA hydrogels here produced, presented valuable characteristics that cannot be found in other fish-derived corneal scaffolds [38,39]. The high capacity to retain water is a critical property in cornea tissue, playing a prominent role in light refraction as well as in ensuring cell survival and proliferation as a nutrient supplier. Marine GelMA hydrogels have been shown to be promising biomaterials meeting these requirements as well as the high transparency that they offer over other cornea substitutes. Moreover, cell encapsulation enables a uniform cell distribution across the construct that otherwise would not be possible when cell seeding is performed after scaffold preparation.

Altogether, our data demonstrated that despite the 15% mGelMA hydrogels presenting greater stability and mechanical properties more closely related to native cornea, the biological performance suggested that the 10% mGelMA hydrogels were more suitable to sustain cellular viability and proliferation. Moreover, hydrogels loaded with 0.5 AA induced a higher expression of corneal stroma ECM specific marker, demonstrating the positive effect of its incorporation into the system. Therefore, the 10% mGelMA hydrogels loaded with 0.5 AA were shown to be the constructs with the most promising potential for corneal stroma regenerative applications.

## 5. Conclusions

In this study, a novel scaffold produced with materials generated from by-products of marine origin was constructed in an attempt to mimic the extracellular matrix of the native corneal stroma. Hydrogels composed of methacrylated gelatin, extracted from codfish skin, were successfully produced by photocrosslinking and loaded with ascorbic acid, revealing good swelling, dimensional stability, and optical properties, as well as good mechanical handling. Human keratocytes loaded into hydrogels were viable and metabolically active. The addition of ascorbic acid enhanced the biological activity of the cells, contributing to the rebuilding of the corneal stroma. The hydrogels appear to have potential to be used as stroma-regeneration-inducing materials, requiring future in vivo studies for a deeper performance evaluation.

## Data Availability

The data that support the findings of this study are available from the corresponding author, upon reasonable request.

## Supplementary Materials

The following supporting information can be downloaded at: https://www.mdpi.com/article/10.3390/bioengineering10010062/s1, Figure S1: Immunofluorescence micrograph of control mGelMA hydrogels (10% mGelMA (A) and 15% mGelMA (B)) stained with anti-rabbit and anti-goat secondary antibodies only. Nuclei were stained with DAPI (blue). Scale bar are 100 µM.
