# Supplementary material for: Cell-Laden Marine Gelatin Methacryloyl Hydrogels Enriched with Ascorbic Acid for Corneal Stroma Regeneration
Source: Bioengineering (Basel). 2023 Jan 4;10(1):62. doi: 10.3390/bioengineering10010062 (PMC9854711; doi:10.3390/bioengineering10010062)
Supplement: Supplementary file 1 [file bioengineering-10-00062-s001.zip › bioengineering-2106821-SI.pdf]

Supplementary material

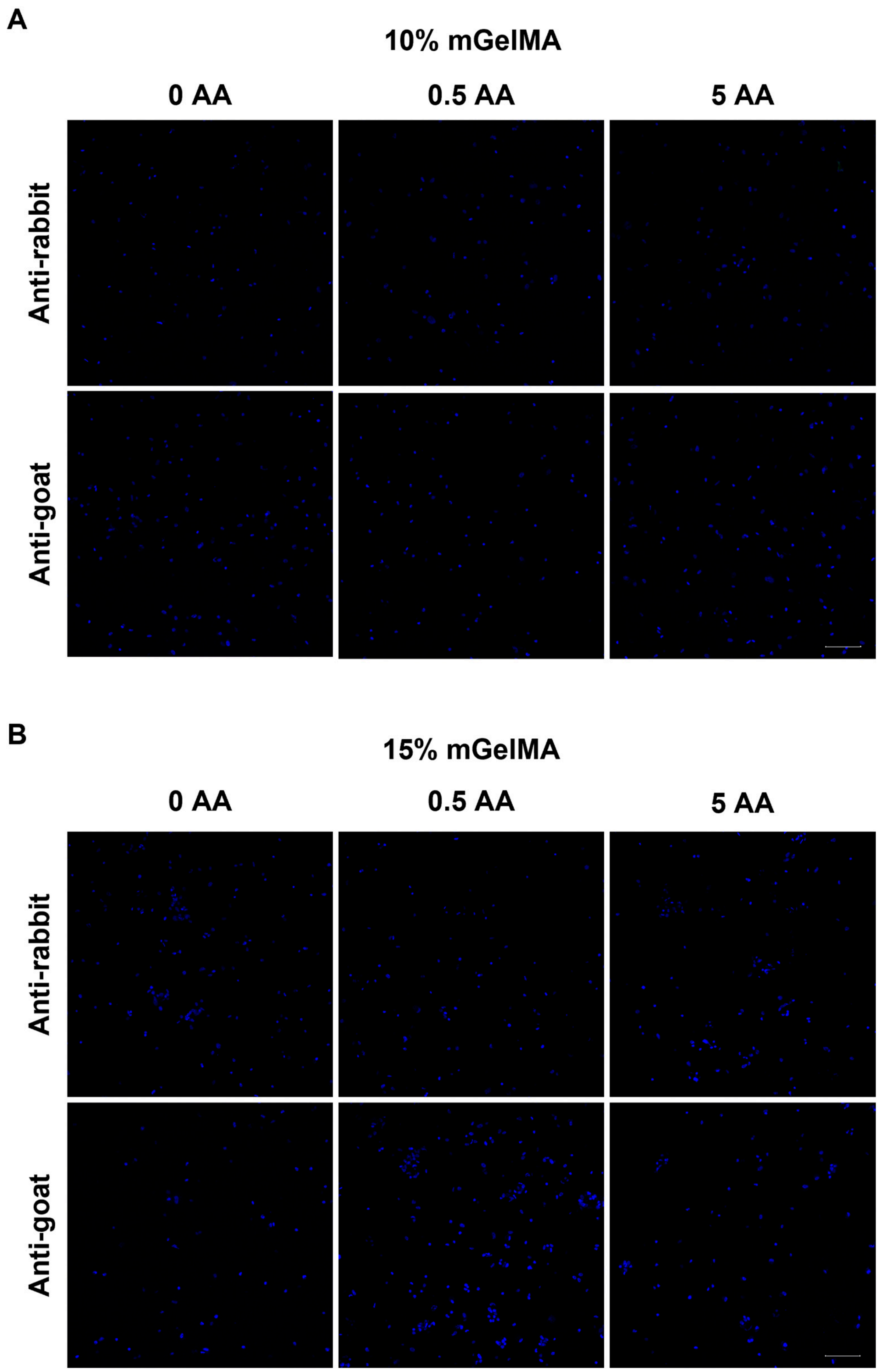

Figure S1. Immunofluorescence micrograph of control mGelMA hydrogels (10% mGelMA (A) and 15% mGelMA (B)) stained with anti-rabbit and anti-goat secondary antibodies only. Nuclei were stained with DAPI (blue). Scale bar are 100 µm.
